# 1,3-Dimethylol-5,5-dimethylhydantoin: MAK-Begründung, Nachtrag

**DOI:** 10.34865/mb644058kskd11_1ad

**Published:** 2026-03-30

**Authors:** Andrea Hartwig, MAK Commission

**Affiliations:** 1 Institut für Angewandte Biowissenschaften, Abteilung Lebensmittelchemie und Toxikologie, Karlsruher Institut für Technologie (KIT) Adenauerring 20a, Geb. 50.41 76131 Karlsruhe Deutschland; 2 Ständige Senatskommission zur Prüfung gesundheitsschädlicher Arbeitsstoffe, Deutsche Forschungsgemeinschaft, Kennedyallee 40, 53175 Bonn, Deutschland. Weitere Informationen: Ständige Senatskommission zur Prüfung gesundheitsschädlicher Arbeitsstoffe | DFG

**Keywords:** 1,3-Dimethylol-5,5-dimethylhydantoin, Nase, oberer Atemtrakt, Formaldehydabspalter, Kanzerogenität, Reizwirkung, Sensibilisierung, Keimzellmutagenität, Strukturanalogie, Luft, air

## Abstract

The German Senate Commission for the Investigation of Health Hazards of Chemical Compounds in the Work Area (MAK Commission) has re-evaluated 1,3-dimethylol-5,5-dimethylhydantoin [6440-58-0] with regard to its carcinogenicity and germ cell mutagenicity classification, its ability to be absorbed through the skin, its sensitization potential and whether an occupational exposure limit value (maximum concentration at the workplace, MAK value) can be derived. Relevant studies were identified from a literature search. The substance is a formaldehyde releaser and is expected to undergo hydrolysis in aqueous solution. For this reason, the observed effects are attributed to the hydrolysis products formaldehyde and 5,5-dimethylhydantoin. There are no studies available that investigated the carcinogenicity, toxicity and genotoxicity of 1,3-dimethylol-5,5-dimethylhydantoin in the upper respiratory tract or nose, which are the likely target organs. The substance has genotoxic potential in vitro, presumably due to the release of formaldehyde. Formaldehyde was classified in Carcinogen Category 4 because it induces tumours in nasal tissue at concentrations that exceed its detoxification capacity. As a formaldehyde releaser, the substance could likewise be classified in Carcinogen Category 4. However, because it is not possible to derive a MAK value for 1,3-dimethylol-5,5-dimethylhydantoin, the substance has been assigned to Carcinogen Category 2 and given the footnote “Prerequisite for Category 4 in principle fulfilled, but insufficient data available for the establishment of a MAK or BAT value”. As there are no data for the systemic bioavailability of 1,3-dimethylol-5,5-dimethylhydantoin and the formaldehyde released by hydrolysis in tissues, there is no experimental evidence that the formaldehyde reaches the germ cells. Therefore, 1,3-dimethylol-5,5-dimethylhydantoin has been classified in Category 3 B for germ cell mutagens. 1,3-Dimethylol-5,5-dimethylhydantoin has skin sensitizing potential and the substance has therefore been designated with “Sh“. Skin contact is not expected to contribute significantly to systemic toxicity.

**Table:** 

**MAK-Wert**	**–**
**Spitzenbegrenzung**	**–**
	
**Hautresorption**	**–**
**Sensibilisierende Wirkung (2002)**	**Sh**
**Krebserzeugende Wirkung (2025)**	**Kategorie 2^a)^**
**Fruchtschädigende Wirkung**	**–**
**Keimzellmutagene Wirkung (2025)**	**Kategorie 3 B**
	
**EKA**	**–**
	
Synonyma	1,3-Bis(hydroxymethyl)-5,5-dimethylhydantoin 1,3-Bis(hydroxymethyl)-5,5-dimethyl-2,4-dioxoimidazolidin DMDM-Hydantoin
Chemische Bezeichnung (IUPAC-Name)	1,3-Bis(hydroxymethyl)-5,5-dimethyl-2,4-imidazolidindion
CAS-Nr.	6440-58-0
Formel	Strukturformel von 1,3-Dimethylol-5,5-dimethylhydantoin. 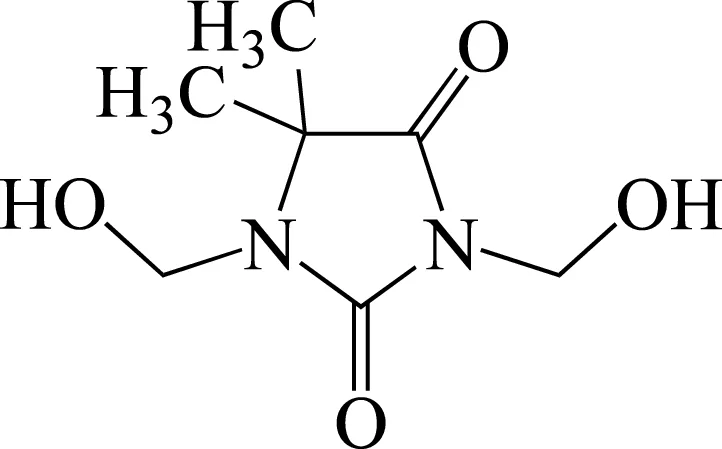
	C_7_H_12_N_2_O_4_
Molmasse	188,19 g/mol
log K_OW_	–2,9 (NCBI 2025)
Wasserlöslichkeit	773 g/l (NCBI 2025)
Schmelzpunkt	102–104 °C (NCBI 2025)
Dampfdruck bei 25 °C	1,2 × 10^–7^ hPa (EU Methode A.4; ECHA 2013)
	
Hydrolysestabilität	OECD-Prüfrichtlinie 111: pH-Wert-abhängige Hydrolyse, bei 35 ± 2 °C und pH 9 schnelle Hydrolyse mit Halbwertszeit (HWZ) < 1 h, bei pH 7 HWZ 10,7 h, bei pH 4 keine Hydrolyse (ECHA 2013). Aus einem Molekül 1,3-Dimethylol-5,5-dimethylhydantoin entstehen zwei Moleküle Formaldehyd und ein Molekül 5,5-Dimethylhydantoin (CAS-Nr. 77-71-4)

^a)^ Voraussetzung für Kategorie 4 prinzipiell erfüllt, aber Daten für MAK- oder BAT-Wert-Ableitung nicht ausreichend

Hinweis: Formaldehydabspalter

Bisher liegen eine Begründung zur Sensibilisierung (Greim 2002) und eine Evaluierung aller Endpunkte (Greim 2004) vor. Es erfolgt eine Neubewertung der Daten zur Formaldehydabspaltung nach aktuellem Vorgehen der Kommission (DFG 2025). Unter diesem Aspekt werden in diesem Nachtrag nur die bewertungsrelevanten Endpunkte re-evaluiert.

Eine Biozid-Registrierung wurde eingereicht, aber bisher keine Daten veröffentlicht (ECHA 2024).

## 1 Allgemeiner Wirkungscharakter

Der vermutlich kritische Effekt von 1,3-Dimethylol-5,5-dimethylhydantoin ist die kanzerogene und reizende Wirkung von freigesetztem Formaldehyd am Atemtrakt. Eine 52,6- bis 55%ige wässrige Lösung wirkt bei einmaliger Applikation beim Kaninchen nicht haut- und leicht augenreizend. 1,3-Dimethylol-5,5-dimethylhydantoin wirkt beim Menschen kontaktsensibilisierend. In vitro ist der Stoff mutagen in Mauslymphomzellen. In bakteriellen Mutagenitätstests wurde nur in den Salmonella-Stämmen TA98 und TA100 ein fraglich positives Ergebnis erhalten. Zwei UDS-Tests sind inkongruent. Eine klastogene Wirkung wird sowohl in Anwesenheit als auch in Abwesenheit eines exogenen metabolischen Aktivierungssystems im Chromosomenaberrationstest beobachtet. Die durchgeführten Untersuchungen zur Genotoxizität der Substanz in vivo (Tests auf Induktion von DNA-Strangbrüchen und DNA-Crosslinks in Testeszellen der Ratte mittels alkalischer Elution und von Mikronuklei im Knochenmark der Maus) liefern keine Hinweise auf genotoxische Wirkungen der Substanz nach oraler Applikation (Greim 2004). Es liegen keine Untersuchungen zur kanzerogenen Wirkung vor.

## 2 Wirkungsmechanismus

1,3-Dimethylol-5,5-dimethylhydantoin ist ein Formaldehydabspalter.

## 3 Toxikokinetik und Metabolismus

Daten nach inhalativer oder oraler Aufnahme liegen nicht vor.

Nach 72-stündiger epikutaner Applikation von 0,1 ml einer Lösung von [5-^14^C]-1,3-Dimethylol-5,5-dimethylhydantoin (Konzentration n. a.) auf die Rückenhaut von Ratten wurden etwa 90 % der verabreichten Radioaktivität wiedergefunden, davon mehr als 98 % an der Applikationsstelle. Die Resorption lag damit bei 2 % (Greim 2004).

Modellrechnungen mit IH SkinPerm v2.04 (Tibaldi et al. 2014) und Fiserova-Bergerova et al. (1990) ergeben für eine gesättigte Lösung unter Standardbedingungen (1 h Expositionsdauer, 2000 cm^2^ Hautfläche) eine perkutane Aufnahme von 55 mg (0,29 mmol) bzw. 191 mg (1,01 mmol) 1,3-Dimethylol-5,5-dimethylhydantoin. Bei vollständiger Freisetzung von Formaldehyd (Molverhältnis 2:1) ist mit einer Freisetzung von 0,58 mmol (17,4 mg) bzw. 2,02 mmol (60,6 mg) zu rechnen.

## 4 Erfahrungen beim Menschen

### Allergene Wirkung

1,3-Dimethylol-5,5-dimethylhydantoin wirkt kontaktsensibilisierend. Entsprechende Studien sind in Greim (2002) dargestellt.

## 5 Tierexperimentelle Befunde und In-vitro-Untersuchungen

### Wirkung auf Haut und Schleimhäute

#### Haut

Eine 55%ige wässrige Lösung wirkte nach einmaliger Applikation beim Kaninchen leicht hautreizend (Greim 2004).

In einer Untersuchung nach Prüfrichtlinie EPA OPP 81-5 wurde jeweils drei männlichen und drei weiblichen Weiße-Neuseeländer-Kaninchen auf die geschorene Haut (6 cm^2^) 0,5 ml einer 52,6%igen wässrigen Lösung von 1,3-Dimethylol-5,5-dimethylhydantoin aufgetragen und vier Stunden lang semiokklusiv exponiert. Danach wurde der Bereich mit Wasser abgewaschen. Der primäre Reizindex (24, 48 und 72 Stunden) betrug 0,73 für Erytheme und 0,2 für Ödeme (k. w. A.). Beide Effekte waren nach sieben Tagen reversibel und die Substanz wurde von den Registranten als nicht hautreizend eingestuft (ECHA 2013).

#### Auge

Eine 55%ige wässrige Lösung von 1,3-Dimethylol-5,5-dimethylhydantoin wirkte nach einmaliger Applikation beim Kaninchen augenreizend (Greim 2004).

In einer Untersuchung nach Prüfrichtlinie EPA OPP 81-4 wurde jeweils drei männlichen und drei weiblichen Weiße-Neuseeländer-Kaninchen 0,1 ml einer 52,6%igen wässrigen Lösung von 1,3-Dimethylol-5,5-dimethylhydantoin in ein Auge instilliert und vier Tage nachbeobachtet. Die Reizwerte (24, 48 und 72 Stunden) für Trübung der Cornea und für die Iris betrugen 0, die Reizwerte für Rötung der Konjunktiven und Tränenfluss betrugen 0,5 bzw. 0,3 (k. w. A.). Beide Effekte waren nach vier Tagen reversibel und die Substanz wurde von den Registranten als minimal reizend beurteilt und nicht als augenreizend eingestuft (ECHA 2013).

### Reproduktionstoxizität

In zwei unveröffentlichten Studien, die in einem Übersichtsartikel zitiert sind, wurden an Kaninchen bei dermaler Applikation bis zur jeweils höchsten Dosis von 413 bzw. 1,32 mg/kg KG und Tag keine Fehlbildungen festgestellt. Jedoch fehlen im Übersichtsartikel detaillierte Angaben zur Entwicklungstoxizität (Greim 2002).

### Genotoxizität

#### In vitro

1,3-Dimethylol-5,5-dimethylhydantoin war in der Mehrzahl der durchgeführten Mutagenitätstests an Bakterien nicht mutagen. Mit den Salmonella-Stämmen TA98 und TA100 traten fraglich positive Ergebnisse auf. Zwei UDS-Tests an Rattenhepatozyten lieferten inkongruente Ergebnisse. Ein Chromosomenaberrationstest an CHO-Zellen und ein TK^+/–^-Mutationstest an L5178Y-Mauslymphomzellen waren in An- und Abwesenheit eines metabolischen Aktivierungssystems positiv. Im Mauslymphomtest wurde die Koloniegröße nicht bestimmt (Greim 2004).

#### In vivo

Tests auf Induktion von DNA-Strangbrüchen und DNA-Crosslinks in Testeszellen der Ratte mittels alkalischer Elution sowie ein Mikronukleustest am Knochenmark der Maus lieferten keine Hinweise auf genotoxische Wirkungen nach oraler Applikation von 1,3-Dimethylol-5,5-dimethylhydantoin (Greim 2004).

### Kanzerogenität

Hierzu liegen keine Daten vor.

## 6 Bewertung 

Das grundlegende Vorgehen zur Bewertung eines Arbeitsstoffes ist der MAK- und BAT-Werte-Liste zu entnehmen (DFG 2025).

Kritische Effekte sind die kanzerogene und lokal reizende Wirkung des Hydrolyseprodukts Formaldehyd.

**Krebserzeugende Wirkung. **Es liegen keine Untersuchungen zur krebserzeugenden Wirkung von 1,3-Dimethylol-5,5-dimethylhydantoin vor. 1,3-Dimethylol-5,5-dimethylhydantoin selbst zeigt eine genotoxische Wirkung in vitro, aber nicht in vivo, eine mögliche genotoxische Wirkung am wahrscheinlichen Zielgewebe Nase (wie bei Formaldehyd) ist jedoch nicht untersucht.

Das Hydrolyseprodukt 5,5-Dimethylhydantoin ist in Fütterungsstudien weder kanzerogen an Sprague-Dawley-Ratten (höchste Dosis 1000 mg/kg KG und Tag) noch an CD-1-Mäusen (höchste Dosis 300 mg/kg KG und Tag) (ECHA 2012).

Die lokale Kanzerogenität des Hydrolyseprodukts Formaldehyd hingegen ist ausführlich dokumentiert (Greim 2000; Hartwig 2010). Formaldehyd ruft Nasentumoren hervor, jedoch erst bei Konzentrationen, die die Entgiftungskapazitäten des Nasengewebes überschreiten. Daher ist Formaldehyd in Kanzerogenitäts-Kategorie 4 eingestuft.

Aufgrund der lokalen kanzerogenen Wirkung von Formaldehyd könnte 1,3-Dimethylol-5,5-dimethylhydantoin in Analogie zu Formaldehyd in Kanzerogenitäts-Kategorie 4 eingestuft werden. Da jedoch kein MAK-Wert für 1,3-Dimethylol-5,5-dimethylhydantoin abgeleitet werden kann, wird der Stoff der Kanzerogenitäts-Kategorie 2 zugeordnet und erhält die Fußnote „Voraussetzung für Kategorie 4 prinzipiell erfüllt, aber Daten für MAK- oder BAT-Wert-Ableitung nicht ausreichend“.

**MAK-Wert. **Es liegen keine Inhalationsstudien mit 1,3-Dimethylol-5,5-dimethylhydantoin am Menschen oder am Tier vor, aus denen ein MAK-Wert abgeleitet werden kann.

Der Stoff hat nach OECD-Prüfrichtline 111 bei 35 ± 2 °C und pH-Wert 7 eine Halbwertszeit von 10,7 h, das bedeutet eine mäßig schnelle Hydrolyse (ECHA 2013). Wegen des Fehlens genauer Daten zur freigesetzten Formaldehyd-Menge in der Lunge bei Inhalation wird als Worst-Case-Abschätzung davon ausgegangen, dass 1,3-Dimethylol-5,5-dimethylhydantoin beim Auftreffen im Atemtrakt sofort vollständig zu Formaldehyd und 5,5-Dimethylhydantoin hydrolysiert und der Abbau des entstehenden Formaldehyds langsamer erfolgt als dessen Bildung. Beim Auftreffen im Atemtrakt ist durch die Hydrolyse daher mit einer inhalationstoxischen Wirkung durch Formaldehyd zu rechnen.

Das Hydrolyseprodukt 5,5-Dimethylhydantoin ist in einer Studie nach OECD-Prüfrichtlinie 405 an Kaninchen nicht augenreizend (ECHA 2012). Formaldehyd hat einen MAK-Wert von 0,3 ml/m^3^ (0,37 mg/m^3^), der vor der lokalen kanzerogenen Wirkung schützt und aus der augenreizenden Wirkung abgeleitet ist (Greim 2000; Hartwig 2010). Sein Dampfdruck bei 25 °C beträgt 5185 hPa (OECD 2002).

Der Dampfdruck von 1,3-Dimethylol-5,5-dimethylhydantoin (1,2 × 10^–7^ hPa; ECHA 2013) zeigt, dass der Stoff als Aerosol vorliegt. Für Aerosole ist durch die Impaktierung im Atemtrakt mit einer stärkeren Wirkung als die durch dampfförmigen Formaldehyd verursachte zu rechnen, wie mit einem anderen Formaldehydabspalter, zu dem eine Inhalationsstudie vorliegt, gezeigt wurde (siehe Begründung N,N′,N′′-Tris(β-hydroxyethyl)hexahydro-1,3,5-triazin; Hartwig und MAK Commission 2023).

Ein MAK-Wert in Analogie zu Formaldehyd kann daher nicht abgeleitet werden. Da auch keine NOAEC aus einer Inhalationsstudie vorliegt, kann für 1,3-Dimethylol-5,5-dimethylhydantoin kein MAK-Wert festgesetzt werden. Eine Spitzenbegrenzung entfällt.

Bei Anwendung in verdünnten wässrigen Lösungen sollte mit einer hydrolytischen Spaltung zu Formaldehyd gerechnet und daher der MAK-Wert für Formaldehyd (Greim 2000; Hartwig 2010) eingehalten werden.

**Keimzellmutagene Wirkung. **1,3-Dimethylol-5,5-dimethylhydantoin ist mutagen im TK^+/–^-Test an Mauslymphomzellen. In Mutagenitätstests an Bakterien war der Stoff nicht mutagen bzw. mit den Salmonella-Stämmen TA98 und TA100 traten uneinheitliche Ergebnisse auf. UDS-Tests an Rattenhepatozyten verliefen ebenfalls uneindeutig. Eine klastogene Wirkung wurde in einem Chromosomenaberrationstest an CHO-Zellen in An- und Abwesenheit eines metabolischen Aktivierungssystems beobachtet. Tests auf Induktion von DNA-Strangbrüchen und DNA-Crosslinks in Testeszellen der Ratte sowie ein Mikronukleustest am Knochenmark der Maus liefern keine Hinweise auf genotoxische Wirkungen nach oraler Applikation von 1,3-Dimethylol-5,5-dimethylhydantoin (Greim 2004). Untersuchungen an Keimzellen liegen mit 1,3-Dimethylol-5,5-dimethylhydantoin nicht vor.

Formaldehyd ist in Kategorie 5 für Keimzellmutagene eingestuft. Dies bedeutet, dass bei Einhaltung des MAK-Wertes von 0,3 ml/m^3^ nur ein sehr geringer Beitrag zum genetischen Risiko für den Menschen zu erwarten ist (Greim 2000).

1,3-Dimethylol-5,5-dimethylhydantoin könnte zwar in Analogie zu Formaldehyd in die Kategorie 5 für Keimzellmutagene eingestuft werden, jedoch kann für 1,3-Dimethylol-5,5-dimethylhydantoin kein MAK-Wert aufgestellt werden.

Da Daten zur systemischen Bioverfügbarkeit des 1,3-Dimethylol-5,5-dimethylhydantoins und dem durch Hydrolyse freigesetzten Formaldehyd fehlen, liegt kein experimenteller Beleg vor, dass der freigesetzte Formaldehyd in aktiver Form die Keimzellen erreicht. Daher wird 1,3-Dimethylol-5,5-dimethylhydantoin in Kategorie 3 B für Keimzellmutagene eingestuft.

**Fruchtschädigende Wirkung. **Da kein MAK-Wert abgeleitet wird, entfällt die Zuordnung zu einer risikobasierten Schwangerschaftsgruppe. Da weder valide Daten zur Entwicklungstoxizität noch Hinweise auf eine mögliche entwicklungstoxische Wirkung vorliegen, lässt sich ein entsprechender Verdacht (Gruppe B (Verdacht)) nicht begründen.

**Hautresorption. **Die perkutane Resorption bei der Ratte ist mit etwa 2 % nach 72 Stunden epikutaner Applikation grundsätzlich sehr gering und die Hydrolyse unter Freisetzung von Formaldehyd erfolgt bei pH-Werten unter 7 sehr langsam (HWZ 10,7 Stunden) oder gar nicht (pH 4). Darüber hinaus ist auch die akute Letalität nach epikutaner Applikation sehr gering (LD_50_ > 10 000 mg/kg KG) und eine 4-wöchige epikutane Applikation von 440 mg/kg KG und Tag beim Kaninchen führte zu keiner systemischen Wirkung (Greim 2004).

Aus den Berechnungen zur möglichen Hautresorption unter Standardbedingungen (1 h Expositionsdauer, 2000 cm^2^ Hautfläche, gesättigte Lösung) lässt sich eine perkutane Aufnahme von etwa 17,4–60,6 mg Formaldehyd abschätzen. Unter Annahme einer konstanten Resorptionsrate und, im Sinne einer Worst-Case-Betrachtung, raschen Hydrolyse des 1,3-Dimethylol-5,5-dimethylhydantoins werden demnach maximal 1,3 mg Formaldehyd/1,25 min (mittlere Halbwertszeit des Formaldehyds im Blut; Kaden et al. 2010) resorbiert bzw. freigesetzt. Der physiologisch bedingte Formaldehydspiegel im Blut des Menschen beträgt etwa 2–3 mg/l (10–15 mg in 5 l Blut frei und reversibel gebunden) (Heck et al. 1985). Die transdermale Aufnahme in den letzten sechs Halbwertszeit-Intervallen führt im Fließgleichgewicht zu einer im Blut zirkulierenden Formaldehydmenge (frei und gebunden) von (1,3 + 0,7 + 0,35 + 0,18 + 0,09 + 0,05) mg = 2,7 mg. Diese Erhöhung der Formaldehyd-Gesamtmenge im Blut liegt im Variationsbereich der physiologischen Belastung, sodass 1,3-Dimethylol-5,5-dimethylhydantoin nicht mit „H“ markiert wird.

**Sensibilisierende Wirkung. **Positive Epikutantestbefunde bestätigen die kontaktallergene Wirkung von 1,3-Dimethylol-5,5-dimethylhydantoin beim Menschen. Daher erfolgt, auch aufgrund der Eigenschaft von 1,3-Dimethylol-5,5-dimethylhydantoin Formaldehyd freizusetzen, weiterhin eine Markierung mit „Sh“. Zur sensibilisierenden Wirkung auf die Atemwege gibt es nach wie vor keine Daten und es erfolgt deshalb weiterhin keine Markierung mit „Sa“.
